# Effect of an Eco-Friendly Cuminaldehyde Guanylhydrazone Disinfectant on Shiga Toxin Production and Global Transcription of *Escherichia coli*

**DOI:** 10.3390/toxins14110752

**Published:** 2022-11-02

**Authors:** Yan Wang, William M. Hart-Cooper, Reuven Rasooly, Michelle Qiu Carter, William J. Orts, Yongqiang Gu, Xiaohua He

**Affiliations:** 1 Western Regional Research Center, Agricultural Research Service, United States Department of Agriculture, 800 Buchanan St., Albany, CA 94710, USA; 2 State Key Laboratory of Infectious Disease Prevention and Control, National Institute for Communicable Disease Control and Prevention, Chinese Center for Disease Control and Prevention, 155 Changbai, Beijing 102206, China

**Keywords:** disinfectant, *Escherichia coli*, reversible antimicrobial guanylhydrazone, Shiga toxin-producing *E. coli*, transcriptome analysis

## Abstract

Antimicrobials have been important medicines used to treat various infections. However, some antibiotics increase the expression of Shiga toxin (Stx). Also, the pervasive use of persistent antibiotics has led to ecotoxicity and antibiotic resistance. In this study, a newly developed broad-spectrum and reversible antibiotic (guanylhydrazone disinfectant) was evaluated for its antibiotic activity and effects on Stx production and global transcription of bacteria. No Stx induction was observed in 25 Shiga toxin-producing *E. coli* (STEC) isolates treated with a sublethal concentration of the guanylhydrazone. A differential gene expression study comparing two guanylhydrazone-treated to non-treated *E. coli* strains indicated that the expression of a group of stress-responsive genes were enhanced. The Kyoto Encyclopedia of Genes and Genomes (KEGG) enrichment analysis revealed that guanylhydrazone treatment significantly downregulated the pathways of ribosome and flagellar assembly in both pathogenic and non-pathogenic strains and differentially regulated some pathways essential for bacteria to maintain cell shape and gain survival advantage in two strains. In addition, upregulation of antibiotic resistant genes related to the multidrug efflux system and virulence genes coding for colibactin, colicin, and adhesin was observed in strains treated with the disinfectant. The knowledge obtained in this study contributes to our understanding of the mode of this disinfectant action and facilitates our effort to better use disinfectants for STEC treatments.

## 1. Introduction

*Escherichia coli* are a large and diverse group of bacteria found in environments, foods, and human and animal intestines. Although most strains of *E. coli* are harmless, some can cause severe public health threats. Shiga toxin-producing *E. coli* (STEC) are a group of prevalent foodborne pathogens that cause more than 265,000 illnesses each year in the United States alone, and the symptoms of the illness ranges from mild diarrhea to bloody diarrhea and life-threatening hemolytic uremic syndrome (HUS) (https://ndc.services.cdc.gov, accessed on 30 September 2022). Treatment of STEC infection with antibiotics is under debate due to the combination of antibiotic-induced Shiga toxin (Stx) production and emerging antibiotic resistance [1,2].

To better control and manage STEC infections, novel and improved interventions are needed. Existing commercial cationic disinfectant quats (e.g., benzalkonium chloride, chlorhexidine), which are used to sanitize or sterilize surfaces of medical and food-processing equipment, are composed of kinetically inert C-C and C-N bonds. These inert bonds are resistant to biodegradation and can result in high environmental persistence, pollution, and danger to water supplies, human health, and agriculture via antibiotic resistance [3,4]. To address these problems, scientists at the USDA–ARS Western Regional Research Center developed eco-friendly disinfectants constructed with reversible hydrazone C=N-NR bonds that link together low-toxicity subcomponent molecules [5]. Unlike relatively inert C-C and C-N bonds, the hydrazone linkage enables sufficient stability for protection against pathogens at low levels of active ingredients, e.g., ~0.1–0.01 wt%. After disposal and dilution to sub-ppm levels in wastewater, the hydrazone bond hydrolyzes, affording subcomponents of smaller magnitudes that are less hazardous than the active hydrazone. This dissociation process is described by experimental association constants (*K*_a_) in water, which range from ~10^4^–10^6^ M^−1^ [6]. Kinetic dissociation rates are typically acid-mediated, with half-lives of hours to months, depending on test conditions, such as pH and temperature [7,8]. These reversible hydrazone disinfectants have good bactericidal effect, low toxicity, less environmental persistence, and lower potential for fostering resistance. However, our knowledge of bacterial cellular responses under exposure to these disinfectants is lacking.

Biological mechanisms of disinfection vary by disinfectants, including interrupting biosynthesis of biological molecules (nucleic acids, proteins, lipids, and carbohydrates), damaging cell membranes, interfering with enzyme systems and metabolisms etc. Understanding the underlying process in microbes when exposing to disinfectants is critical for the improvement of disinfection effectiveness and process innovation. In this study, we aimed to examine the effect of a reversible guanylhydrazone disinfectant on the Stx production by STEC strains, and we compared the gene expression of nonpathogenic *E. coli* and pathogenic STEC exposed to a sublethal concentration of the disinfectant using transcriptome sequencing analysis. Based on its high potency and low hazard subcomponents, the cuminaldehyde guanylhydrazone was selected for study in this work. Identification of common genes that are differentially regulated by disinfectants found in both pathogenic and nonpathogenic strains could lead to a better understanding of the effects of this disinfectant on bacterial growth, which is important for future development of improved interventions.

## 2. Results and Discussion

### 2.1. Susceptibility of E. coli Strains to the Guanylhydrazone and the Sublethal Dose for Treatment

**Figure 1:**
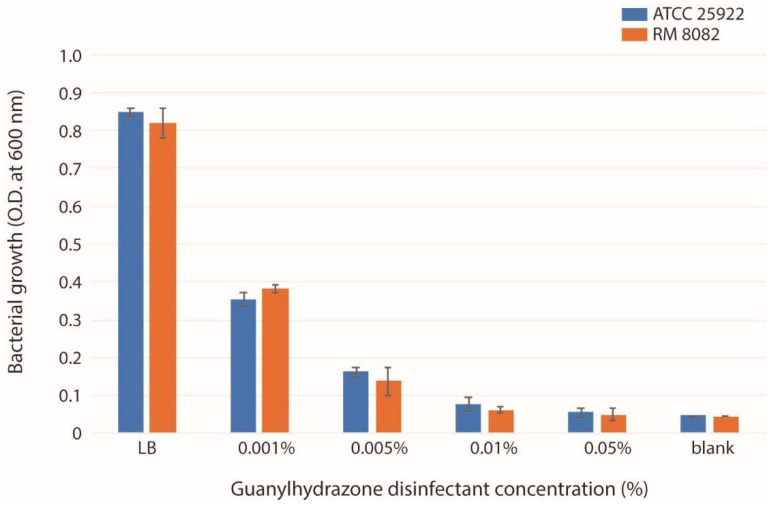
Growth states of *E. coli* strains ATCC25922 and RM8082. The O.D. values were obtained from overnight cultures of strains ATCC25922 and RM8082 grown in LB broth, LB broth containing four different concentrations of the guanylhydrazone disinfectant (0.001%, 0.005%, 0.01%, and 0.05%), and LB broth without bacteria (blank). The results represent the mean ± SD of three independent experiments.

The antibacterial effect of the chemical disinfectant guanylhydrazone was evaluated by treating liquid cultures of two *E. coli* strains (RM8082 and ATCC25922) with four different concentrations of the disinfectant: 0.001%, 0.005%, 0.01%, and 0.05%. The treatment with 0% of the disinfectant in LB was used as a negative control. After overnight treatment, both *E. coli* strains were completely inactive at concentrations of 0.01% and 0.05% and their OD values were equal to the blank control. The OD values of both strains showed 2- and 4-fold reduction, respectively, after incubation overnight in LB containing 0.001% and 0.005% of the disinfectant when compared with their negative controls (LB only) (Figure 1). To better understand the mode of this disinfectant action and the ability of bacteria to survive the treatment, 0.005% guanylhydrazone was used as the sublethal concentration to treat the bacteria.

### 2.2. Effect of the Guanylhydrazone on the Production of Stx1

Antibiotics are one of the most highly utilized medication classes for bacterial infections, but not all antibiotics work against every infection. For instance, there is no widely accepted antibiotic treatment for STEC infections because certain antibiotics induce Stx production, resulting in a higher risk of HUS development [9]. To investigate the effect of guanylhydrazone on Stx production, 25 in-house STEC strains containing *stx1* gene were tested (Table 1).

**Figure 2:**
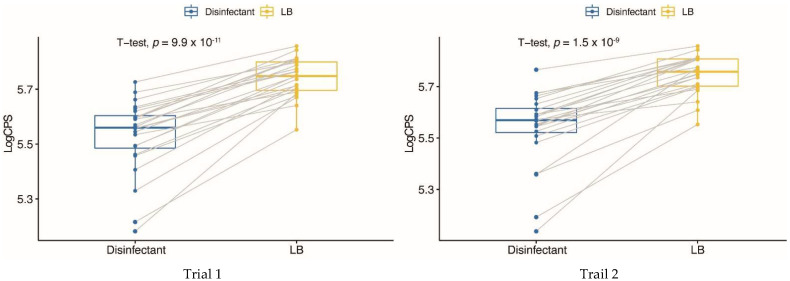
The effect of the guanylhydrazone disinfectant on the production of Stx1. Twenty-five *stx1*-positive *E. coli* strains were grown in LB with and without a sublethal concentration of the disinfectant. The relative amount of Stx1 (cps) was measured by sandwich ELISAs using mAb Stx1-2 as a capture antibody and Stx1 pAb as a detection antibody. Each result represents the mean of triplicates from one experiment, and two independent experiments were performed on two different days.

The luminescence signals (cps-counts per second) obtained from ELISAs were used to determine the relative abundance of Stx1 produced by each strain. It was found that the Stx1 levels varied significantly among 25 Stx1-producing *E. coli* strains in the presence or absence of the guanylhydrazone. However, cells treated with the sublethal concentration of guanylhydrazone (0.005%) all produced significantly less Stx1 (*p* < 0.01) than cells from the same strain without guanylhydrazone treatment (Figure 2), suggesting that the guanylhydrazone did not induce Stx1 production. Therefore, the guanylhydrazone could be useful for the management of STEC infections due to its unique function as a bactericide that does not induce Stx production.

### 2.3. Global Gene Expression Profiles of Two Strains Exposed to the Guanylhydrazone

To understand the biological responses triggered by the chemical disinfectant, two bacterial strains with complete genome sequences available were selected. RNA samples from two strains (non-pathogenic ATCC25922 and pathogenic RM8082) treated and non-treated with the chemical disinfectant were extracted. Changes in gene expression of the two strains after treating with the disinfectant were analyzed. Based on whole genome sequencing data, strain ATCC25922 contains two plasmids, both of which were predicted to code 92 coding DNA sequences (CDSs) and one chromosome containing 4648 CDSs. A total of 1956 differentially expressed genes (DEGs) (42% of CDSs) were identified in the chromosome, including 787 upregulated genes and 1169 downregulated genes in treated cells (Table S1). In plasmid 1, eight DEGs were identified and all of them were upregulated. Three of the eight DEGs were predicted to code phage structure proteins. While in plasmid 2, 10 DEGs were identified, of which five genes were upregulated and five were downregulated. It is worth noting that the five upregulated genes in plasmid 2 were successive, among which the gene *mccB* and the gene coding ubiE/COQ5 methyltransferase family protein were drastically increased with a log2 fold change (log2FC) of 3.82 and 3.21, respectively. Based on whole genome sequencing data, strain RM8082 harbors a chromosome and four plasmids. There were 749 (15%), 35 (23%), 29 (25%), 12 (11%), and 5 (10%) DEGs (% of CDSs) observed in the chromosome and plasmid 1 to plasmid 4, respectively. In the chromosome of RM8082 treated cells, 383 DEGs were upregulated and 366 DEGs were downregulated (Table S1). The majority of DEGs on the plasmids were upregulated. In plasmid 1, 29 genes were upregulated, and 6 genes were downregulated. In plasmid 2, plasmid 3, and plasmid 4, all DEGs were upregulated. Although the majority of DEGs on the plasmids have not been well-characterized and were annotated as hypothetical proteins, many stress-responsive genes were drastically upregulated, including SOS mutagenesis associated genes, such as *dinI*, *umuD*, *umuC1* and *umuC2* in plasmid 2, colicin coding genes, *cia,* in plasmid 2, *cba* (colicin B), *cma* (colicin M) in plasmid 3, and alleviating oxidative stress associated gene *katG* in plasmid 1. The induction of stress-responsive genes is a common mechanism for bacteria to cope with unfavorable environmental conditions. For example, the SOS response is a cellular DNA repair mechanism that plays an important role in bacterial resistance to antibiotics that cause DNA damages [10]. Colicin is normally produced to out-compete other bacteria under limited resources, while the alleviated hydro peroxidase could be there to cope with hydrogen peroxide.

### 2.4. Shared Gene Expression Patterns in Two E. coli Strains

**Figure 3:**
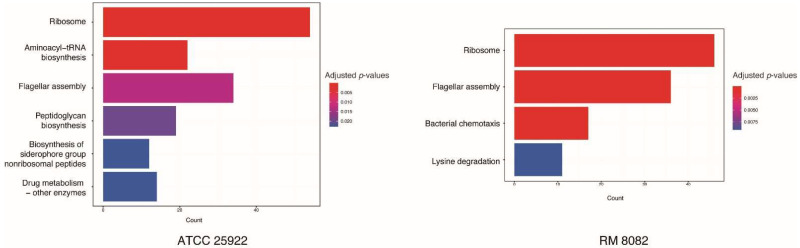
KEGG Pathway Enrichment analysis performed on DEGs. Bar plots showed the significantly enriched KEGG pathways when the strains ATCC25922 (**left**) and RM8082 (**right**) responded to a sub-lethal dose of the disinfectant. *Y*-axis represents pathways, *X*-axis represents the amount of the stress-related mRNAs enriched in KEGG pathways; an adjusted *p* < 0.01 was used as the threshold to select KEGG terms. KEGG, Kyoto Encyclopedia of Genes and Genomes.

To identify pathways that were upregulated or downregulated in response to the chemical disinfectant treatment for the two *E. coli* strains, the Kyoto Encyclopedia of Genes and Genomes (KEGG) pathway enrichment analyses were performed. KEGG Pathway enrichment analysis revealed that the ribosome and flagellar assembly were significantly enriched with downregulated genes following exposure to the sublethal concentration of the disinfectant for both *E. coli* strains (Figure 3). It was found that 54 and 46 genes coding for the large (rpl/rpm) and small (rps) ribosomal subunits, and 33 and 36 flagella assembly associated genes were downregulated in strains ATCC25922 and RM8082, respectively. Among these genes, *flgB* and *flgC,* involving in the assembly of the rod structure of flagellar basal body, were found to be downregulated most significantly. The genes involved in the C ring (*fliG*, *fliM* and *fliN*), P-ring (*flgA*), L-ring (*flgH* and *flgI*), and M ring (*fliF*) exhibited downregulation as well. In addition, the flagellar motor complex coding genes, *motA* and *motB*, in strain RM8082 were also downregulated.

These results suggest that most genes involved in ribosome and flagellar assembly pathways shared between pathogenic and non-pathogenic bacteria and their expression were repressed in the presence of the disinfectant. This is not surprising because both bacterial ribosomes and flagella are essential to *E. coli* and are highly conserved and evolutionarily stable. Guanylhydrazone derivatives have been used as antifungal agents and DNA was found to be their initial target of action [11]. Results from this study indicated that the treatment of bacterial strains ATCC25922 and RM8082 with the guanylhydrazone disinfectant resulted in downregulation of 54 and 46 genes coding for the ribosomal subunits, suggesting that ribosomes could be the initial or secondary target of the guanylhydrazone. Several major classes of antibiotics were found to inhibit protein synthesis by binding specifically to the 30S [12,13] or 50S ribosomal subunits [14]. Bacterial resistance to these drugs is often acquired by mutations leading to amino acid substitution in ribosomal proteins, enzymatic inactivation of the antibiotic, impermeability, or efflux of the antibiotic [15]. Most *E. coli* strains have flagella that help them to move, form biofilm, adhere to surfaces, and invade host cells [16,17]. Over 60 structural and regulatory genes are required for flagellum assembly and function [18]. The guanylhydrazone downregulated the expression of 33 and 36 genes responsible for flagella assembly in the two strains, respectively, and the most significant reduction happened for genes coding for the rod structure proteins of the flagellar basal body, *FlgB* and *FlgC*. Giacomucci et al. observed that treatment of *V. cholerae* with polymyxin B significantly reduced the quantity of cell-attached flagellin and cell mobility, which was correlated with a reduction of the biofilm formation [19]. The downregulation of flagella assembly by the sublethal concentration of guanylhydrazone disinfectant found in this study suggests that this disinfectant could use flagellum as one of initial targets to affect bacterial movement and biofilm formation, resulting in eventual cell death.

### 2.5. Differences in Gene Expression between Two E. coli Strains

Expression analysis also revealed different patterns of gene expression between the two *E. coli* strains (Figure 3). In addition to the ribosome and flagella assembly pathways, strain ATCC25922 was also enriched with downregulated genes involved in four pathways: aminoacyl-tRNA biosynthesis, peptidoglycan biosynthesis, biosynthesis of siderophore group non-ribosomal peptides, and drug metabolism. The function of aminoacyl-tRNA biosynthesis is to precisely match amino acids with tRNAs containing the corresponding anticodon, and plays a central role in protein biosynthesis. A total of 22 DEGs in this pathway were observed, including 21 downregulated genes and one upregulated gene (*lysS*). The downregulated genes were involved in the biosynthesis of all kinds of amino acid, except for the biosynthesis of serine and threonine. Two *lysS* genes (locus tag: DR76_RS09035 and DR76_RS11400) coding for the lysine-tRNA ligase were upregulated and downregulated, respectively. This data further indicated that guanylhydrazone may inactivate bacteria through blocking protein synthesis. The biosynthesis of peptidoglycan is critical for forming cell walls, which provides bacterial cells with both structural support and protection from mechanical and osmotic stress. In this pathway, 18 genes were downregulated and only 1 gene was upregulated. Among the downregulated genes, gene *murA* (which catalyzes the first committed step in peptidoglycan biosynthesis) showed the highest downregulation level with the log2FC of −2.96, followed by gene *mrcA* coding for the penicillin-binding protein 1A with the log2FC of −2.35. The gene *mtgA* coding for the peptidoglycan transglucosylase (which catalyzes glycan chain elongation) was observed to be slightly upregulated with a log2FC of 1.30. In the biosynthesis of siderophore group non-ribosomal peptides pathway, seven genes (*entA–F* and *entH*) involved in the biosynthesis of the siderophore enterobactin were found to be downregulated. While four genes (*irp1*, *irp2*, *ybtE*, and *ybtS*) involved in the biosynthesis of yersiniabactin showed slight upregulation with a log2FC ranging from 1.27 to 1.43. Drug metabolism is the major route for all living organisms to eliminate poisonous compounds. In strain ATCC25922 treated with the disinfectant, 10 downregulated and 4 upregulated DEGs were identified in the pathway of drug metabolism. All 10 downregulated DEGs were found to be associated with biosynthesis of nucleoside/nucleotide. Of the 4 upregulated DEGs, genes *gst* and *katG* (associated with protecting against oxidative stress) had log2FCs of 3.63 and 3.10, respectively.

In addition to the downregulation of ribosome and flagella assembly pathways in strain RM8082, the strain was also enriched with downregulated genes associated with the bacterial chemotaxis pathway (Figure 3). Among 17 DEGs, 5 were also involved in the pathway of flagella assembly. A ten-gene operon (*motAB-cheAW-tar-tap-cheRBYZ*) was found to be downregulated with a log2FC ranging from 2.72 to 3.55. It was reported that bacterial motility and chemotaxis were required for many pathogenic species to colonize and invade a host [20,21]. The downregulation of chemotaxis pathways in strain RM8082, but not in ATCC25922, suggests that the effect of guanylhydrazone on chemotaxis may be specific to pathogenic bacteria. In contrast, genes involved in the pathway of lysine degradation were upregulated in strain RM8082. The expression level of the five-gene operon (*csiD-lhgO-gabDTP*) was drastically increased with a log2FC ranging from 4.9 to 5.8. It is not clear if this differential expression is preferentially tied up with pathogenic bacteria.

### 2.6. Expression of Resistance Genes and Virulence Genes in E. coli Challenged with the Guanylhydrazone

In strain ATCC25922 treated with the disinfectant, 44 antibiotic resistance genes (ARGs) and 224 virulence genes were identified using ABRicate (version 1.0.1) against the comprehensive antibiotic resistance database (CARD) and virulence genes database (ecoli_vf), respectively. Among the 44 ARGs, 25% of them (11) were significantly downregulated and 11.4% (5) were significantly upregulated (Table S1, yellow highlights). The upregulated ARGs, including *mdtH*, *mdtG*, *mdtM evgA*, and *gadX* were mainly involved in the multidrug efflux system. Among the 224 virulence genes, 34.8% of them (78) were downregulated and 17.4% (39) were upregulated (Table S1, green highlights). Except for *clbN*, *clbP*, and *clbS*, all genes located in the *pks* gene island, which is composed of 19 encoding colibactin genes (*clbA-S*), were upregulated. In addition, our data revealed that disinfectant treatment also induced the expression of *pgaA–D* genes, which are responsible for synthesis, modification, and translocation of polysaccharide. The biofilm adhesin poly-β-1,6-N-Acetyl-d-Glucosamine was also upregulated.

In strain RM8082, there were 42 ARGs and 172 virulence genes identified. Among the 42 ARGs, 2 (4.8%) were downregulated and 3 (7.1%) were upregulated (Table S2, yellow highlights). As in strain ATCC25922, the upregulated ARGs, including *gadWX* and *marA*, were associated with the multidrug resistance efflux pump. Among the 172 virulence genes, 49 (28.5%) were downregulated and 6 (3.5%) were upregulated (Table S2, green highlights). Three upregulated virulence genes, including *cma* (colicin M), *cba* (colicin B), and *clpF*, were on plasmids. The gene *eaeH* (notable for encoding an outer membrane protein—adhesin—that is necessary to produce A/E lesions (attaching and effacing)) was slightly induced by the disinfectant with a log2FC of 1.50.

## 3. Conclusions

We have confirmed the inhibitory effect of the guanylhydrazone disinfectant on *E. coli* growth with a minimum inhibitory concentration of 0.01%, and shown that the disinfectant did not induce Stx1 production in 25 STEC strains tested. Differentially expressed genes were identified in two *E. coli* strains treated with the disinfectant. The KEGG enrichment analysis revealed 2 downregulated pathways associated with ribosome and flagella assembly in both pathogenic and non-pathogenic strains, and 5 other downregulated pathways essential for drug metabolism, chemotaxis, and the biosynthesis of proteins, cell walls, and siderophores in one or the other strain in response to the disinfectant. In addition, treatment with the disinfectant enhanced the expression of antibiotic resistance genes responsible for the multidrug resistance efflux pump, and virulence genes encoding colibactin, colicin and adhesin, which reflect cell strategies for survival and persistence. Results from this study suggested that the guanylhydrazone disinfectant likely suppressed *E. coli* growth through inhibiting protein synthesis and cell movement. However, the detailed mode of action of the guanylhydrazone disinfectant remains vague, further investigation is needed to improve the application efficiency and develop new antibiotic agents to combat the rapid emergence of antibiotic resistance in pathogenic bacteria.

## 4. Materials and Methods

### 4.1. Cuminaldehyde Guanylhydrazone

The cuminaldehyde guanylhydrazone was prepared as described previously [22].

### 4.2. Bacterial Strains and Growth Conditions

Bacterial strains used to assess the effect of the disinfectant on Stx production are listed in Table 1. These strains were obtained from in-house stocks. They were revived from stock cultures that kept in Luria-Bertani (LB) containing 20% glycerol and frozen at −80 °C. The sublethal dose of the guanylhydrazone against *E. coli* strains were determined by growing bacteria in LB broth containing four different concentrations of the guanylhydrazone: 0.001%, 0.005%, 0.01% and 0.05%. The effect of the guanylhydrazone on Stx production were investigated in 25 Stx1-producing *E. coli* strains, and strain ATCC25922 was used as a negative control. Bacterial strains were grown from single colonies in 1 mL LB medium at 37 °C overnight. Each overnight culture was then diluted 1:50 in LB and LB with sublethal dose of the disinfectant (0.005%), and continued to grow overnight at 37 °C with shaking. Bacterial cultures with similar number of cells from disinfectant-treated samples (60 µL) and non-treated samples (20 µL, plus 40 µL of LB to bring the final volume to 60 µL) were then treated with an equal volume of phosphate Bacterial Protein Extraction Reagent (B-PER, Pierce Biotechnology, Rockford, IL, USA) for 1 h at 37 °C. Following centrifugation at 13,000× *g* for 10 min at 4 °C, 100 µL of bacterial culture supernatants were collected and used for detection of Stx1 by enzyme-linked immunosorbent assay (ELISA). Each treatment was repeated twice on two different days.

### 4.3. ELISA for Stx Production

Sandwich ELISAs were performed as previously described [23]. Briefly, 96-well black NUNC Maxisorp flat bottom plates (Thermo Scientific, Waltham, MA, USA) were coated with 100 µL/well of 1 µg/mL of the capture antibody, Stx1-2 [24] and incubated at 4 °C overnight. After overnight incubation, the plates were washed two times with 0.02 M Tris buffered saline with 0.9 % NaCl, pH 7.4, and 0.05% Tween-20 (TBST), blocked in 5% non-fat dry milk (NFDM)-TBST for an hour at 37 °C, and then incubated with samples for 1 h at 37 °C. The plates were washed with TBST six times and incubated with 100 ng/mL of detection antibody, Stx1 pAb [23], in NFDM-TBST. Goat anti-rabbit HRP (Promega, Madison, WI, USA), 10 ng/mL in NFDM-TBST, was added as a secondary antibody after washing six times with TBST. Plates were developed with 100 µL SuperSignal West Pico Chemiluminescent Substrate (Thermo Scientific, Waltham, MA, USA), and luminescence counts per second (CPS) were read on a Victor 3 plate reader (Perkin Elmer, Shelton, CT, USA) for 0.1 s. Each treatment was performed in triplicate and repeated twice on two different days.

### 4.4. Statistical Analysis

To determine if the disinfectant induced Stx production in STEC strains, 25 *Stx1*-positive *E. coli* strains were tested for *Stx1* production in the presence and absence of the disinfectant by ELISA. Statistical analyses were performed using paired *t*-test to compare the amounts of *Stx1* produced by bacteria treated and non-treated with disinfectant. Statistical significance was declared when *p* < 0.01. All statistical analyses were performed with the R-package, version 4.1.2. (https://www.R-project.org, accessed on 30 September 2022).

### 4.5. RNA Isolation and RNA Sequencing

The complete genome sequences of strains RM8082 (CP043825.1–CP043829.1 with one chromosome and 4 plasmids) and ATCC25922 (CP009072–CP009074 with one chromosome and 2 plasmids) were available in the NCBI database, therefore they were selected for transcriptome analysis. Bacterial total RNA was extracted using Qiagen RNeasy mini kit (Germantown, MD, USA) and quantified using a Nanodrop (Wilmington, DE, USA). The RNA quality was monitored with an Agilent 2100 Bioanalyzer (Agilent Technologies, Santa Clara, CA, USA). After removal of ribosomal RNA using Ribominus Transcriptome Isolation Kit (Invitrogen life technologies, Carlsbad, CA, USA), the samples were converted to cDNA prior to Illumina sequence library construction using a ScriptSeq v2RNA-Seq Library Preparation Kit (Epicenter Biotechnologies, Madison, WI, USA). The quality, quantity, and size distribution of the Illumina libraries were determined using the Agilent 2100 Bioanalyzer. The resulting libraries were combined equally (100 pM each) and submitted to Genomic Core at Michigan State University for Illumina HiSeq2000 run.

### 4.6. Transcriptomic Analysis

Analysis of RNA sequencing data, including raw reads filtering, reads alignment, and reads counting, were all implemented in CLC Genomics Workbench v.8.1 (Qiagen, Hilden, Germany). After counting the reads of each gene, calculation and normalization of differential expression levels were conducted using the DESeq2 R-package. Significantly differentially expressed genes were determined when the fold change values are greater than 2.0 (the cutoff value) and an adjusted *p*-value < 0.01. The DEGs were then used for Kyoto Encyclopedia of Genes and Genomes (KEGG) enrichment analyses. Fisher’s exact test was applied to perform enrichment analysis using the R-package clusterProfiler version 4.0 (Bioconductor). Pathways with a q value ≤ 0.05 that were significantly enriched in DEGs in the comparison between the treatment and control were analyzed with the KEGG database.

## Figures and Tables

**Table 1:** Strains used in this study.

Strain	Serotype	*stx1*	Origin
RM2367	O157:H7	+	Human
RM6649	O157:H7	+	Human
RM7370	O111	+	Water
RM7375	O26	+	Human
RM7543	O157:H7	+	Human
RM7927	O26	+	Water
RM7958	O113	+	Cow feces
RM8082	O121	+	Cow feces
RM8385	O103	+	Cow feces
RM8426	O26	+	Water
RM8876	O145	+	Water
RM9306	O145	+	Cow feces
RM9322	O111	+	Water
RM9413	O45	+	Cow feces
RM9882	O103	+	Cow feces
RM9907	O111	+	Feral pig
RM9917	O145	+	Feral pig
RM9975	O111	+	Crow
RM10061	O103	+	Feral pig
RM10408	O103	+	Crow
RM10817	O26	+	Cow feces
RM12788	O111	+	Human
RM13506	O45	+	Human
RM13508	O103	+	Human
RM13752	O45	+	Cow feces
ATCC25922	O6	-	ATCC

## Data Availability

The data presented in this study are available in supplementary material here.

## Supplementary Materials

The following supporting information can be downloaded at: https://www.mdpi.com/article/10.3390/toxins14110752/s1, Table S1: Differentially expressed genes (DEGs) identified in strain ATCC25922 and Table S2: Differentially expressed genes (DEGs) identified in strain RM8082.
